# The influence of obstructive sleep apnea syndrome on anthropometric parameters at 12 months after laparoscopic sleeve gastrectomy

**DOI:** 10.1038/s41598-021-85192-8

**Published:** 2021-03-11

**Authors:** Laura Mihalache, Dimitrie Siriopol, Lidia Iuliana Arhire, Sergiu Pădureanu, Cristina Preda, Daniela Boișteanu, Dragoș Scripcariu, Silvia Cusai, Adrian Covic

**Affiliations:** 1 Diabetes, Nutrition and Metabolic Diseases Department, “Grigore T. Popa” University of Medicine and Pharmacy, Str. Universității 16, 700115 Iași, Romania; 2 “Sf. Spiridon” Clinical Emergency Hospital, Iași, Romania; 3 Nephrology Department, “Grigore T. Popa” University of Medicine and Pharmacy, Iași, Romania; 4 Endocrinology Department, “Grigore T. Popa” University of Medicine and Pharmacy, Iași, Romania; 5 Pneumology Department, “Grigore T. Popa” University of Medicine and Pharmacy, Iași, Romania; 6 Surgery Department, “Grigore T. Popa” University of Medicine and Pharmacy, Iași, Romania

**Keywords:** Endocrine system and metabolic diseases, Metabolic disorders, Respiratory tract diseases

## Abstract

The aim of this study was to assess the influence of obstructive sleep apnea syndrome (OSAS) on the change in anthropometric parameters and body composition, in patients undergoing laparoscopic sleeve gastrectomy (LSG). This prospective study included patients undergoing LSG who had pre-operative polysomnography data and were also evaluated at six and 12 months after surgery. All patients included also had whole body composition analysis data before surgery and at six and 12 months after surgery. The results are presented in comparison between patients with and without OSAS. We included 73 patients in the analysis with a mean ± SD age and body mass index (BMI) of 40.3 ± 10.9 years and 45.4 ± 6.3 kg/m^2^, respectively. As compared to the baseline levels, at 6 months there was a significant decrease in BMI, weight, waist circumference, serum glucose and HbA1c. At 12 months there was no further decrease as compared to the 6 months levels, irrespective of OSAS status. We observed a significant decrease at 6 months in percentage of fat, in both types of patients. However, as compared to the 6 months levels, at 12 months the percent fat had a significant decrease only in patients without OSAS (− 4.6%, 95% CI − 7.6 to − 1.7%) and not in those with OSAS (− 2.2%, 95% CI − 4.5 to 0.2%). In our study, patients with OSAS showed a similar decrease in different anthropometric parameters as those without OSAS after LSG. However, at 12 months of follow-up there was a significant decrease in the percent fat only in patients without OSAS.

## Introduction

Obesity is now considered a chronic disease with a worldwide epidemic evolution, associated with numerous complications that can reduce the quality of life and life expectancy of the patients, but tremendously increase the burden on health systems. Obstructive sleep apnea syndrome (OSAS) is a frequent complication of obesity; conversely, almost all patients with OSAS (96%) have excess weight^1^. In addition, current estimates of the prevalence of OSAS show an evolution which is parallel to that of the obesity epidemic^2,3^.

Obesity predisposes to and is considered a major risk factor for the development and progression of OSAS. The proposed mechanisms are multiple and still incompletely known. Local and systemic inflammation^4^, hormonal disorders with increased susceptibility to stress hormones, catecholamines and testosterone^5,6^, as well as leptin-ghrelin imbalance with increased appetite^7^, metabolic dysfunction with increased insulin resistance^8^ have all been associated with OSAS. Nevertheless, excessive weight represents the major risk factor and weight loss the main “curative” approach.

Unfortunately, weight loss through behavioral, lifestyle, and pharmacological treatment interventions improve OSAS-specific symptoms in the short term, but long-term outcomes are often discouraging. In this context, metabolic surgery may be a more effective long-term strategy, not only for the control of weight and metabolic parameters^9–12^, but also for the remission of OSAS symptoms, with a significantly greater reduction in OSAS severity compared to non-surgical methods^13–15^.

Laparoscopic sleeve gastrectomy (LSG) has been rapidly growing in popularity, which is why there has been a vivid interest to compare it to the *gold-standard* bariatric procedure, which is Roux-en-Y gastric bypass (RYGB)^16^. These comparisons have mainly tackled the weight loss and remission of diabetes^17,18^ and also looked at the possibility to use the same predictors for success in LSG, as for RYGB – for example, the ABCD score for the remission of diabetes^19^. However, there are very few studies who investigated the way OSAS could influence weight loss after LSG^20^, and none which looked particularly at fat loss as measured by dual energy X-ray absorptiometry (DXA).

The aim of this study was to describe the observed relationship between OSAS and body weight and composition changes at six months and one year after LSG.

## Methods

### Patients

We conducted a prospective study in which we included patients who underwent LSG between 2014 and 2018 at the Center for Bariatric Surgery, “Sf. Spiridon” Clinical Emergency Hospital, Iași, Romania. This study was approved by our Institutional Review Board of the “Grigore T. Popa” University of Medicine and Pharmacy, and all the patients included gave informed consent for their participation in the study. This work follows the STrengthening the Reporting of OBservational studies in Epidemiology (STROBE) guidelines for cohort studies^20^.

### Pre-operative assessment

Weight status was evaluated using the classical anthropometric parameters of weight, body mass index (BMI) and waist circumference (WC), and dual energy X-ray absorptiometry (DXA) was used to assess body composition.

We collected blood samples from all patients after a 12 h overnight fast to test for glycemia, glycated hemoglobin (HbA1c), total proteins and albumin, uric acid, lipid profile, CRP, creatinine; the samples were processed on the same day at the hospital laboratory using standard techniques. The metabolic syndrome was defined using the International Diabetes Federation recommendations^21^.

The OSAS was diagnosed by ambulatory or in-hospital cardio-respiratory polysomnography, using a Porti 7 device. Patients were referred to the sleep lab of the 3rd Pneumology Clinic for high pretest suspicion of OSAS, including excessive daytime sleepiness (Epworth scale score of > 10/24). For each patient, a six-channel cardio-respiratory polysomnography was performed, including measurements of nasal airflow via nasal cannula, monitoring of respiratory effort with chest and abdominal band, continuous pulse-oximetry, body position and snoring. The Porti 7 cardiorespiratory polysomnography was performed and manually scored by a trained physician, according to the American Academy of Sleep Medicine standards. Apnea and hypopnea events and the apnea–hypopnea index (AHI) were defined according to the recommendations of the American Academy of Sleep^22^. OSAS was considered absent when AHI was under 5 events/hour.

### Surgery and postoperative assessment

The surgery was carried out by the same surgical team using a standard technique for LSG (a 32-Fr bougie size was used).

We used the recommendations of the American Society for Metabolic and Bariatric Surgery to report on the results on weight evolution after surgery (the outcome reporting standards)^23^. Apart from polysomnography, which was performed only once, prior to surgery, all the other parameters described above were assessed during the preoperative and the 6 months and 12 months postoperative follow-up.

Therefore, *inclusion criteria* were: all patients who gave consent and who undertook preoperative ambulatory or in-hospital cardio-respiratory polysomnography and also were evaluated by dual energy X-ray absorptiometry (DXA) to assess body composition; only these patients were considered for the postoperative follow-up.

### Statistical analysis

Data are presented as mean ± standard deviation, median with interquartile range or number and percent frequency, as appropriate. The comparison between groups was performed using the Chi-square test for categorical variables and the Mann–Whitney test or independent t test for the remaining variables, as appropriate. The normality of the distribution was assessed using the Shapiro–Wilk test.

Time repeated measurements were analyzed using linear mixed models including OSAS status, time, and the OSAS status by time interaction term. Normally distributed continuous variables were assessed through mixed models for repeated measurements, and for non-normally distributed data by penalized quasi-likelihood under restricted maximum likelihood models. All models were adjusted for baseline values. Treatment inferences, effect estimates, and 95% CIs were taken from these models.

As a sensitivity analysis, we also divided the patients with OSAS into two additional groups, according to the metabolic syndrome status, and performed the same analyses as described above.

We considered a P value of less than 0.05 to be significant. All analyses were performed using Stata SE software, version 12 (Stata Statistical Software: Release 12. College Station, TX: StataCorp LP.).

Our primary end-point was the influence of OSAS on fat loss, and secondary end-point was the influence of OSAS on weight loss and metabolic parameters, during the 12 months follow-up after surgery.

## Results

### Baseline characteristics

**Figure 1:**
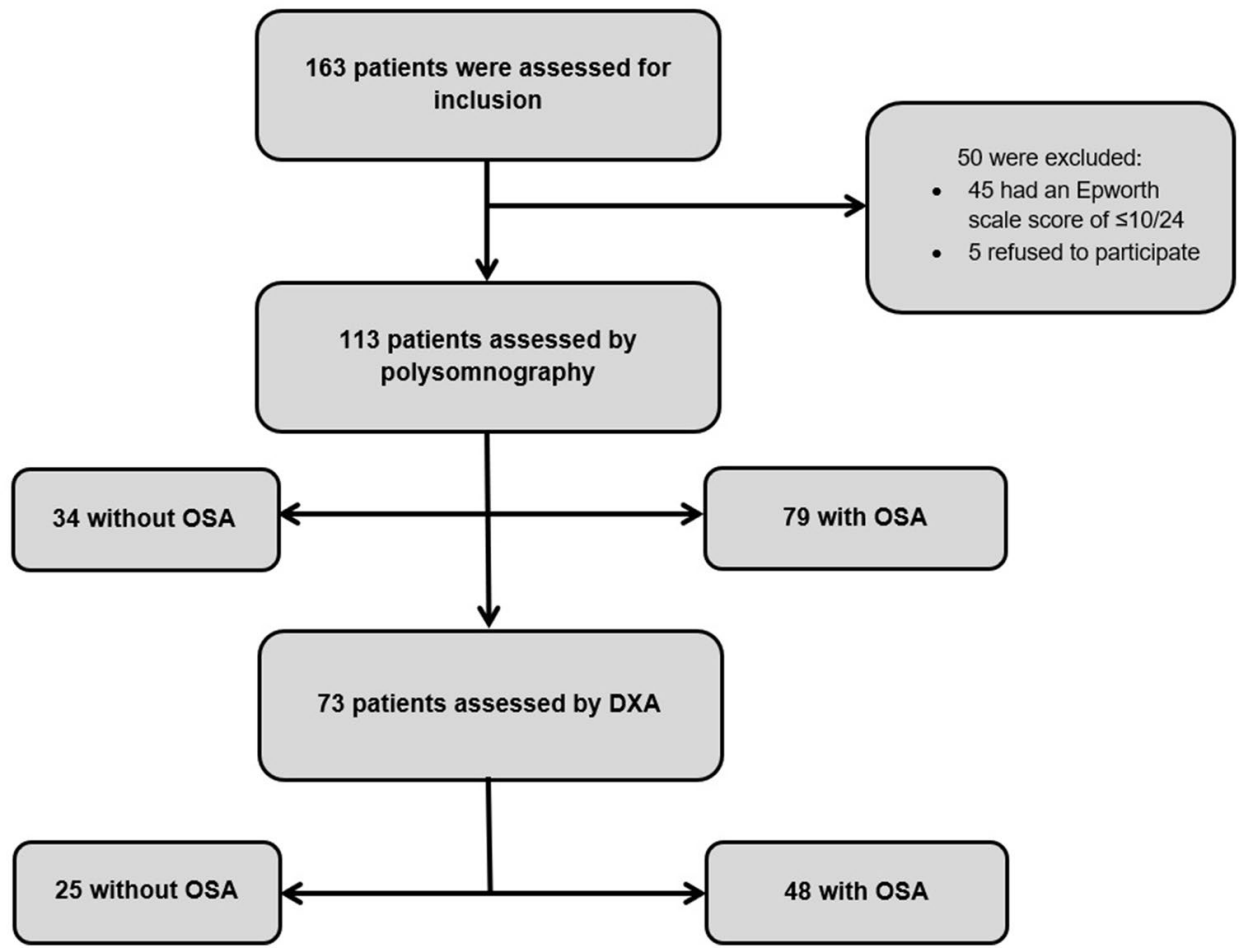
Flow-chart of the study population.

 Our study was carried out on 73 patients (48 with OSAS), as explained in Fig. 1: from the 163 available patients, 45 (27.6%) were excluded because they had an Epworth scale score of ≤ 10/24 and as such weren’t evaluated by polysomnography and an additional 5 (3.1%) refused to participate in the study. DXA analysis was performed at baseline in only 73 patients (due to technical restrictions). Baseline demographic, clinical and biological characteristics of the population are presented in Table 1. At baseline, patients with OSAS were older, with higher waist circumference (WC) and glycemic level.

**Table 1:** Baseline characteristics of the study population.

	Total (N = 73)	No OSA (N = 25)	OSA (N = 48)	*p*
Age, years	40.3 ± 10.9	34.4 ± 8.4	43.3 ± 10.9	** < 0.001**
Male, N (%)	17 (23.3)	4 (16.0)	13 (27.1)	0.29
Diabetes, N (%)	27 (37.0)	8 (32.0)	19 (39.6)	0.52
BMI, kg/m^2^	45.4 ± 6.3	44.6 ± 5.5	45.9 ± 6.7	0.40
Weight, kg	126.8 ± 19.4	122.6 ± 18.7	128.9 ± 19.6	0.19
Waist circumference, cm	127.2 ± 13.0	122.0 ± 11.9	129.9 ± 12.8	**0.01**
SBP, mmHg	133.9 ± 18.5	129.9 ± 12.8	136.0 ± 12.8	0.13
DBP, mmHg	83.2 ± 11.2	81.9 ± 8.2	83.8 ± 12.5	0.45
Hemoglobin, g/dL	13.6 ± 1.4	13.4 ± 1.2	13.7 ± 1.5	0.39
Serum glucose, mg/dL	98.0 (92.0–112.0)	96.0 (85.0–102.0)	102.5 (93.5–122.5)	**0.004**
HbA1c, %	5.5 (5.2–5.9)	5.3 (4.9–5.5)	5.7 (5.3–6.0)	**0.002**
Total cholesterol, mg/dL	204.4 ± 48.4	209.1 ± 42.7	201.9 ± 51.4	0.56
HDL cholesterol, mg/dL	44.9 ± 10.0	47.2 ± 10.2	43.8 ± 9.9	0.17
LDL cholesterol, mg/dL	131.5 ± 34.0	142.4 ± 34.6	125.9 ± 32.7	0.05
Triglycerides, mg/dL	136.0 (99.0–183.0)	113.0 (100.0–165.0)	142.5 (97.0–203.0)	0.33
Uric acid, mg/dL	5.8 ± 1.4	5.5 ± 1.4	5.9 ± 1.4	0.15
Total proteins, g/dL	7.4 ± 0.5	7.4 ± 0.5	7.4 ± 0.5	0.56
Albumin, g/dL	4.4 ± 0.3	4.5 ± 0.3	4.4 ± 0.3	0.37
eGFR, ml/min/1.73 m^2^	103.8 ± 15.3	109.2 ± 13.9	100.9 ± 15.4	**0.03**
CRP, mg/dL	0.8 (0.5–1.1)	0.7 (0.4–0.9)	0.9 (0.6–1.2)	0.09
Fat, Kg	57.1 ± 11.5	54.9 ± 9.4	58.3 ± 12.4	0.23
Percent fat, %	45.2 ± 5.1	44.9 ± 4.7	45.3 ± 5.4	0.46

Data are expressed as mean ± SD, median with IQR, or percent frequency, as appropriate. Bold values are statistically signifcant.

BMI—body mass index; CRP—C reactive protein; DBP—diastolic blood pressure; eGFR—estimated glomerular filtration rate; HbA1c—glycated hemoglobin; HDL—high-density lipoprotein; LDL—low-density lipoprotein; SBP—systolic blood pressure.

### Changes in anthropometric and glucose homeostasis parameters during follow-up

We assessed the changes in BMI, weight, waist circumference, serum glucose and HbA1c at the 6- and 12-months postoperative follow-up. As compared to baseline levels, at 6 months there was a significant decrease in BMI, weight, WC, glucose and HbA1c. At 12 months, BMI levels continued to be lower than the baseline ones, but there was no further decrease as compared to the 6 months levels, irrespective of OSAS status. Similar patterns were noted for weight and waist circumference, glucose and HbA1c (Table 2). The difference between WC of patients with OSAS and patients without OSAS was kept throughout the follow-up period.

**Table 2:** BMI, weight, waist circumference, Fat and Percent Fat, serum glucose and HbA1c levels evolution during the follow-up across the two OSA groups.

	Baseline	6 Months	12 Months	P*	*P*^†^
**BMI, kg/m**^**2**^					
No OSA (N = 25)	44.6 ± 5.5	32.79 (30.02–35.56)	29.19 (25.47–32.9)	< 0.001	0.58
OSA (N = 48)	45.9 ± 6.7	35.13 (32.56–37.7)	33.15 (29.85–36.45)		
*P*^‡^	–	0.24	0.128		
**Weight, kg**					
No OSA (N = 25)	122.6 ± 18.7	89.38 (80.99–97.75)	79.4 (69.42–89.38)	< 0.001	0.71
OSA (N = 48)	128.9 ± 19.6	99.23 (91.81–106.66)	93.7 (83.24–104.16)		
*P*^‡^		0.092	0.076		
**Waist circumference, cm**					
No OSA (N = 25)	122.0 ± 11.9	97.87 (90.63–105.11)	91.89 (82.96–100.82)	< 0.001	0.51
OSA (N = 48)	129.9 ± 12.8	107.78 (102.03–113.53)	105.47 (97.22–113.72)		
*P*^‡^	–	0.034	0.035		
**Fat, kg**					
No OSA (N = 25)	54.9 (50.6–59.1)	33.1 (29.9–36.3)	27.5 (23.6–31.3)	< 0.001	0.45
OSA (N = 48)	58.3 (55.3–61.3)	34.9 (32.3–37.6)	31.7 (28.7–34.7)		
*P*^‡^	–	0.38	0.09		
**Percent Fat, %**					
No OSA (N = 25)	44.9 (42.8–47.2)	35.4 (33.8–36.9)	30.8 (28.8–32.7)	< 0.001	0.02
OSA (N = 48)	45.3 (43.7–46.9)	36.9 (35.6–38.3)	34.8 (33.2–36.3)		
*P*^‡^	–	0.16	0.002		
**Serum glucose, mg/dL**					
No OSA (N = 25)	96.0 (85.0–102.0)	86.53 (82.62–90.44)	88.6 (82.56–94.64)	< 0.001	0.19
OSA (N = 48)	102.5 (93.5–122.5)	90.5 (82.02–98.97)	86.85 (82.15–91.55)		
*P*^‡^	–	0.5	0.64		
**HbA1c, %**					
No OSA (N = 25)	5.3 (4.9–5.5)	5.2 (5–5.3)	5 (4.7–5.3)	< 0.001	0.4
OSA (N = 48)	5.7 (5.3–6.0)	5.4 (5.1–5.7)	5.2 (5–5.3)		
*P*^‡^	–	0.335	0.172		

Data are presented as mean (95%CI) at baseline, and least-squares mean (95%CI) at 6 and 12 months. Analysis was conducted using a mixed model for repeated measures, adjusting for baseline values. Adjusting also for diabetes didn’t change the significance of the results.

*P value for time effect – trend over time in all arms.

^†^*P* value for treatment x time interaction – evaluates if changes in one group are different from the changes in the other group.

^‡^*P* value for comparison between groups at each moment; BMI—body mass index; HbA1c—glycated hemoglobin.

### Changes in body fat

**Figure 2:**
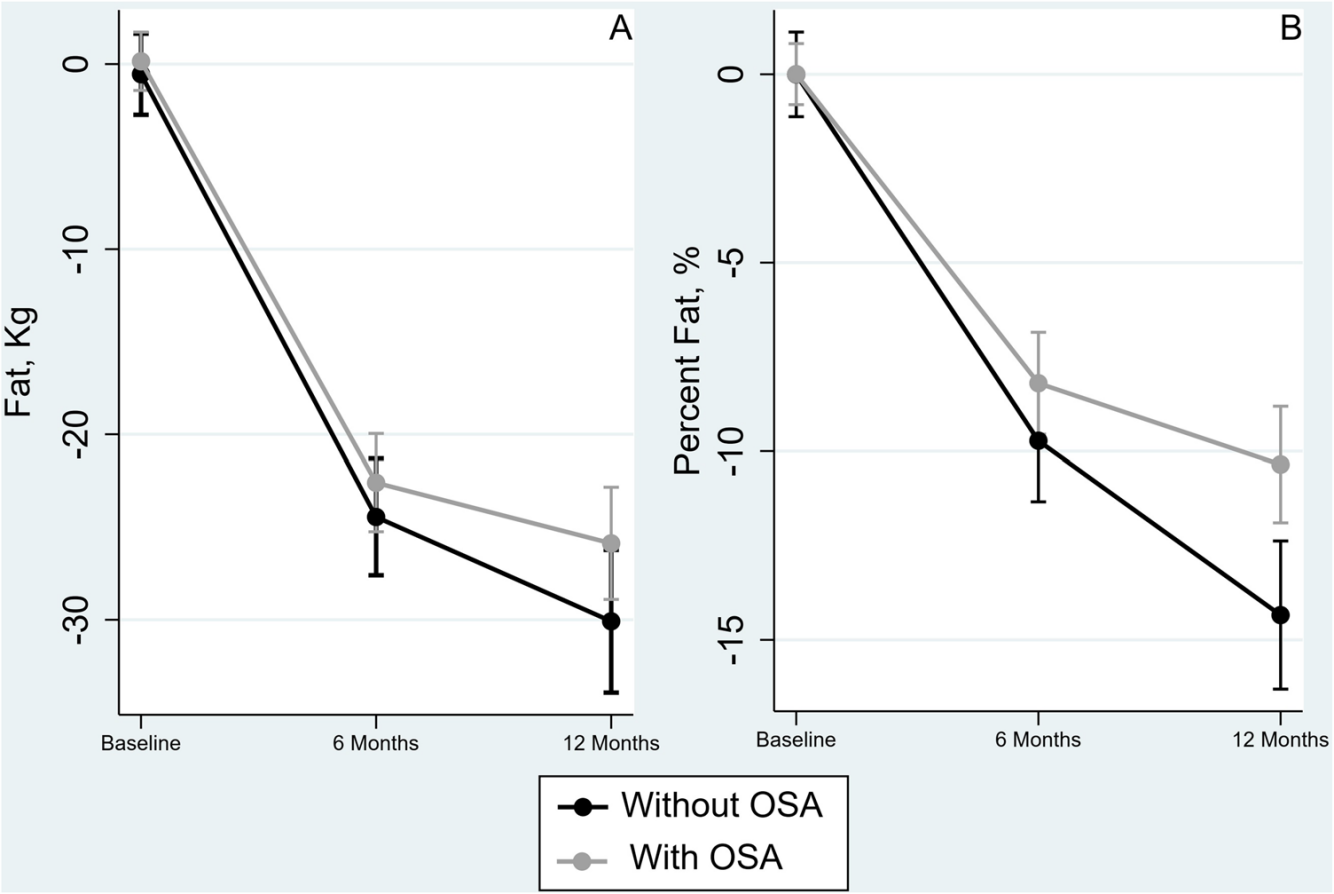
The decrease in body fat (**A**) and percentage of body fat (**B**) at 6 and 12 months after surgery in patients with and without obstructive sleep apnea syndrome (OSAS).

As compared to the baseline levels, there was a significant decrease in fat levels at 6 and 12 months, irrespective of OSAS status; however, there was no significant decrease in fat values at 12 months, as compared to the 6 months values (Table 2 and Fig. 2A).

When we analyzed the change in percentage of fat, as compared to baseline levels, there was a significant decrease at 6 months, in both types of patients (− 9.7%, 95% CI − 12.0 to − 7.4% and − 8.2%, 95% CI − 10.1 to − 6.4% in patients without and with OSAS, respectively). However, as compared to the 6 months levels, at 12 months the percent fat had a significant decrease only in patients without OSAS (− 4.6%, 95% CI − 7.6 to − 1.7%) and not in those with OSAS (− 2.2%, 95% CI − 4.5 to 0.2%) – see Table 2 and Fig. 2B.

### Sensitivity analysis

We further divided the patients with OSAS into two groups, based on the presence of the metabolic syndrome. When performing the same analyses, we didn’t find any difference between these two groups of patients in regard with changes in anthropometric and glucose homeostasis parameters or changes in body fat during the follow-up (data not shown).

## Discussion

To our knowledge, this is the first study to assess the influence of OSAS on body composition, as evaluated by dual energy X-ray absorptiometry (DXA), 12 months after LSG. When describing weight loss after surgery, most studies use classic anthropometric parameters, without mentioning the evolution of different components of body composition. However, one major aim of any weight loss program in patients with morbid obesity is to reduce fat mass while conserving lean mass. Therefore, using the evolution of body composition parameters when assessing predictive factors would be more useful in establishing optimal individualized therapeutic interventions.

Most literature data suggest that surgical treatment provides a more significant and sustained weight reduction compared to non-surgical treatment. Similarly^15^, improving respiratory status is explained by both weight-dependent mechanical mechanisms and metabolic effects independent of the weight status (BRAVE effects—bile flow alteration, reduction of gastric size, anatomical gut rearrangement and altered flow of nutrients, vagal manipulation, enteric gut hormone modulation).

The increased risk of OSAS could hamper weight loss in patients with obesity and metabolic syndrome, as shown by a secondary analysis of a randomized control trial of a nutritional intervention: nearly half of the participants (45.8%) were at increased OSAS risk, and they lost less weight than those without OSAS (1.2% ± 4.2% vs 4.2% ± 5.3%), having a lower chance of losing 5% or more of their weight (24.4% vs. 75.6%)^24^.

The presence of certain characteristics may represent a sign of inappropriate weight loss in post metabolic surgery period. Thus, age, BMI, the presence of diabetes or OSAS are inversely associated with the weight evolution at 1 year postoperative^20,25–29^. As compared with the aforementioned studies our patients were younger^20,26,27^, only Caucasian^29^ and received only the LSG procedure^20^ and these dissimilarities could, at least in part, explain the different results observed in regard to the relationship between weight loss and the OSAS.

The bidirectional relation between obesity and OSAS is well established^2^, but recent data suggest that OSAS determines also cardio-metabolic dysfunction^30,31^. The proposed mechanisms are complex and not fully understood: overstimulation of the sympathetic nervous system, tissue hypoxia, oxidative stress, chronic inflammation and others^32^. A more recent theory describes “lipotoxicity” as a mechanism in which the metabolism of free fatty acids is altered during sleep in patients with OSAS leading to an exaggerated lipolysis, increase in free fatty acids and consequently to a shift in the distribution of adipose tissue from the subcutaneous to visceral site^33^. This pathological mechanism is in line with the recently published results by Dalmar et al.^34^. The authors showed that although patients had a similar reduction in BMI after banding, those with OSAS had an increased risk for cardiovascular events (represented by newly installed stroke, heart failure, myocardial infarction, venous thrombosis, pulmonary embolism) and only a trend towards a higher mortality risk at 3 years post-surgery^34^.

Given these theories and our results, we could select patients with increased cardio-metabolic risk to be included in intensive individualized management programs. Our study shows that patients with severe obesity who underwent LSG had a similar weight reduction regardless of the presence of OSAS, but patients with OSAS lost significantly less fat mass compared to those without OSAS, in the first year after surgery. Therefore, although the evolution of weight and metabolic parameters was favorable, patients with OSAS could retain a higher cardiometabolic risk post-surgery and would need more individualized monitoring.

### Strengths and limitations

The major strength of our study is the extensive and homogeneous evaluation of the patients: all patients were assessed both by cardio-respiratory polysomnography and dual energy X-ray absorptiometry (DXA) and they were all evaluated and treated by the same multidisciplinary team – followed the same protocol of postoperative follow-up, benefited from the same sessions of therapeutic education with the implementation of the same recommendations regarding nutritional intake and physical activity, pre- and post-operative lifestyle. Also, all patients included in the study benefited from CPAP treatment if needed, pre- and postoperatively (we do not have data about their respiratory status post-surgery). One limitation of this study is the relatively short duration of follow-up. However, this is a common duration of follow-up presented in studies and also studies that followed patients for more than five years showed that the most significant changes happen in the first year after surgery^35,36^**.** Nevertheless, this is an ongoing study and we will be able to evaluate further body composition changes in the future years. Another limitation of the study is that the two groups of patients (with and without OSAS) are not matched in terms of age and some metabolic factors, but there were no significant difference preoperatory in terns of BMI, fat mass and fat percent.

#### Conclusion

In our study, patients with OSAS showed a similar decrease in different anthropometric parameters as those without OSAS after LSG. However, at 12 months of follow-up there was a significant decrease in the percent fat only in patients without OSAS.

## Abbreviations

AHI: Apnea–hypopnea index
BMI: Body mass index
DXA: Dual energy X-ray absorptiometry
EBMIL: Excess body mass index loss
EWL: Excess weight loss
LSG: Laparoscopic sleeve gastrectomy
OSAS: Obstructive sleep apnea syndrome
TWL: Total weight loss
WC: Waist circumference
